# Retrograde Intramedullary Nailing for Periprosthetic Supracondylar Fractures of the Femur after Total Knee Arthroplasty

**DOI:** 10.4055/cios.2009.1.4.201

**Published:** 2009-11-25

**Authors:** Hyuk-Soo Han, Kyu-Won Oh, Seung-Baik Kang

**Affiliations:** Department of Orthopaedic Surgery, Seoul National University Boramae Medical Center, Seoul, Korea.

**Keywords:** Femoral fracture, Intramedullary nailing, Total knee arthroplasty, Retrograde, Periprosthetic

## Abstract

**Background:**

Periprosthetic supracondylar fractures of the femur after total knee arthroplasty are not common but are usually difficult to treat due to the advanced age of patients and frequently accompanying osteoporosis. Retrograde intramedullary nailing can be effective in promoting healing of these fractures by providing sufficient stability, but the number of beneficiaries is small due to its limited applicability and the postoperative function has rarely been assessed. This study evaluated the efficacy of retrograde intramedullary nailing for the treatment of periprosthetic supracondylar fractures of the femur using the clinical outcomes.

**Methods:**

Between January 2000 and May 2006, 9 patients (10 knees) with periprosthetic supracondylar fractures of the femur underwent retrograde intramedullary nailing. An open reduction and additional fixation using a shape memory alloy ring were used in 3 of them in whom a closed reduction was not successful. The clinical and radiographic findings were reviewed retrospectively in 7 patients (8 knees), excluding 2 who were unavailable for a follow-up assessment due to death. The mean follow-up period was 39 months (range, 24 to 82 months). The union and alignment of the fracture were assessed radiographically. The postoperative function was evaluated using Sanders' criteria.

**Results:**

Radiographic union was obtained in all patients after an average of 13 weeks (range, 12 to 15 weeks) postoperatively. No postoperative infection, heterotopic ossification and component loosening were observed. After union, the coronal alignment averaged 0.1° valgus (range, 3.6° varus to 2.6° valgus) and the mean sagittal alignment was 1.9° of extension (range, 0.9° of flexion to 6.3° of extension). The mean range of motion was 103° (range, 90° to 120°) postoperatively. At the last follow up, there were 1 excellent, 5 good and 2 fair results according to Sanders' criteria.

**Conclusions:**

With retrograde intramedullary nailing, excellent fracture union and good functional recovery were obtained in patients with periprosthetic supracondylar fractures.

With the aging of the population, more total knee arthroplasties are being performed in patients advanced in age, and periprosthetic fractures are becoming more common due to the increasing postoperative activities.^1^,2) Periprosthetic fractures after total knee arthroplasty are defined as fractures occurring in the femur, tibia and patella and within 15 cm of the joint line or 5 cm of the intramedullary stem.3) These fractures, which were first reported by Hirsh et al. in 1981, are mostly observed near the femoral component and are known to occur in 0.3-2.5% of patients after primary total knee arthroplasty and in 1.6-38% of patients following revision total knee arthroplasty.^4^,5)

The treatment principles for periprosthetic fractures following total knee arthroplasty include maintaining alignment with rigid internal fixation, obtaining bone union and recovering sufficient range of motion of the knee through early exercise. The treatment options include conservative treatment, such as closed reduction and cast immobilization, and surgery, such as open reduction and internal fixation, intramedullary nailing, revision total knee arthroplasty using a longer stem, external fixation and arthrodesis with bone graft.^1^,5) Unfortunately, stable fixation is difficult to achieve in many cases due to the age and accompanying osteoporosis.

Retrograde intramedullary nailing was first introduced for the treatment of supracondylar fractures of the femur in 1991 and attempted in total knee arthroplasty patients in 1994.4) The technique is relatively simple to perform and enhances fracture healing by providing proper stability with minimal soft tissue stripping. However, it can be applied to patients with supracondylar fractures of the femur after total knee arthroplasty only when there is no femoral component loosening, the intercondylar notch is properly open and comminution of the distal femur is sufficiently minor to allow the stable insertion of at least 2 distal interlocking screws. Accordingly, the study population sizes are usually small and the postoperative function has rarely been assessed. In addition, there are concerns regarding the angular deformity or fracture displacement because the location of the entry hole should follow the intercondylar notch of the femoral component. This study was undertaken to analyze the clinical outcomes of retrograde intramedullary nailing for periprosthetic supracondylar fractures of the femur after total knee arthroplasty and examine the efficacy of the procedure with a more than 2 year follow-up.

## METHODS

This study included 9 patients (10 knees), who had been admitted to this hospital between January 2000 and May 2006 for periprosthetic supracondylar fractures of the femur after total knee arthroplasty. Excluding 2 patients who died during the follow-up, 7 patients (8 knees) were reviewed retrospectively for the clinical and radiological assessment. Their mean age was 68 years (range, 65 to 74 years). Six were female and one was male. Each had undergone total knee arthroplasty with a diagnosis of osteoarthritis. The implants employed included LCS (DePuy, Warsaw, IN, USA) in 3 knees, Scorpio CR (Stryker, Allendale, NJ, USA) in 2 knees and Scorpio PS (Stryker) in 3 knees. The cause of the periprosthetic fracture was a fall in all cases. The mean interval from total knee arthroplasty to the development of a fracture was 40.1 months (range, 8 to 123 months). All fractures were Type II according to the classification system of Rorabeck and Taylor6) Fixation was performed using retrograde intramedullary nails (AIM® titanium supracondylar nail, DePuy ACE, Leeds, UK) in all cases.

The compatibility of retrograde intramedullary nails and implanted prostheses was reviewed preoperatively. All operations were performed by the same surgeon and a tourniquet was used. The patients were placed in the supine position on a radiolucent operating table. A rolled drape was placed under the thigh to facilitate 40°-50° of knee flexion and closed reduction was attempted with traction. A skin incision was made using the scar from previous surgery and the intercondylar notch of the femoral component was exposed using the medial parapatellar approach. A pilot hole was made in the intercondylar notch with a drill. A guide wire was placed using c-arm fluoroscopy and the medullary cavity was reamed. A reamer, 12 mm in diameter was also used near the opening. The alignment of the fracture was examined in both sagittal and coronal planes and a minimally invasive open reduction was followed by additional fixation using a shape memory alloy (Bio-smart, Ulsan, Korea) in 3 cases with ≥ 5° of angulation on either the coronal or sagittal planes after the closed reduction. Rigid fixation was obtained with bone cement in 3 cases whose distal interference screw fixation was unsuccessful due to severe comminution of the femoral metaphysis or osteoporosis. From the 1st postoperative day, joint exercise using a continuous passive motion machine was started and weight bearing was allowed when callus formation was visible on the radiographs (at approximately 6th postoperative week).

A postoperative assessment was performed on an outpatient basis at the 4th, 8th, 12th, and 24th postoperative week and then once a year thereafter. Fracture union was assessed based on the radiological and clinical findings. Clinical union was confirmed by bridging callus formation on the anteroposterior and lateral radiographs and pain free weight bearing. Radiological union was defined as the presence of the trabecular or cortical bone across the fracture site. In addition, tibiofemoral alignment (in sagittal and coronal planes) was measured on the simple radiographs. Sanders' criteria was used for a functional assessment7) (Table 1).

## RESULTS

**Fig. 1:**
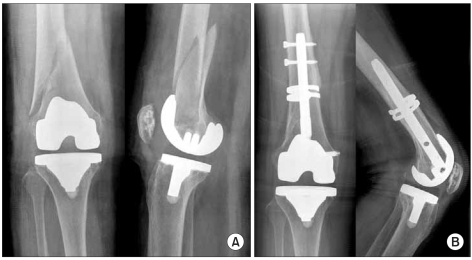
(A) Preoperative X-rays of a periprosthetic fracture of the right knee in a 57-year-old woman. (B) X-rays taken 5 months later show mature callus formation. A short supracondylar nail was used due to femoral anterior bowing.

The mean postoperative follow-up period was 39 months (range, 24 to 82 months). The mean operation time was 65 minutes (range, 55 to 85 minutes). In all cases, clinical union was observed and full weight bearing was achieved at the 13th postoperative week on average (range, 12th to 15th week) (Fig. 1). Radiological union was also noted in all cases on the radiographs taken at 6th postoperative months. After union, the coronal alignment averaged 0.1° of valgus (range, 3.6° of varus to 2.6° of valgus) and the average sagittal alignment was 1.9° of extension (range, 0.9° of flexion to 6.3° of extension). At the last follow-up, the mean range of motion was 103° (range, 90° to 120°). There were 1 excellent, 5 good, and 2 fair results according to the Sanders' criteria (Table 2).

Postoperatively, nerve or vessel damage was not observed, and there were no complications such as an infection or heterotopic ossification. Component loosening did not occur, and no revision was required in any case.

## DISCUSSION

Periprosthetic supracondylar fractures of the femur after total knee arthroplasty are uncommon and difficult to treat. However, their incidence is expected to increase with the aging of the population and the increasing postoperative activities. Although there are many classification methods and treatment algorithms for periprosthetic supracondylar fractures of the femur after total knee arthroplasty, none have been accepted as the 'gold standard'.8) It is important to maintain a range of knee joint motion > 90° and obtain fracture union without component loosening. In addition, the decision regarding the treatment of periprosthetic fractures is difficult to make because it should be based on a variety of factors, such as the stability of an implant in the bone, patient's systematic condition before and after fracture, knee joint condition and alignment of the fracture site.^9^-11) Non-surgical treatments are non-invasive and have a low risk of infection. On the other hand, they restrict elderly patients' mobility, which can also lead to functional impairment, such as myoatrophy and ankylosis, and other complications, such as deep venous thrombosis.^7^,12)

There are a range of surgical procedures performed for stable fixation and early exercise, each having different benefits and weaknesses. Although there are few reports on external fixation, it has been associated with infection risks, difficulty in pin insertion, and refracture due to the stress concentration at the pin insertion site.^5^,9) Revision arthroplasty may bring about a rapid recovery but is very invasive and requires a bone graft for bone loss. Open reduction is advantageous in achieving anatomical reduction because a direct view of the fracture site can be secured during surgery. However, it is likely to result in nonunion or infection due to an invasive incision and soft tissue stripping. In the case of a severely comminuted fracture, fixation of a metal plate and screws are difficult to obtain and postoperative reduction is quite likely to fail.^13^,14) Although a less invasive stabilization system has been described as a more satisfying procedure than the established open reduction techniques in recent reports, a delay in bone union caused by soft tissue stripping is a major problem, and biomechanical studies have shown that retrograde intramedullary nailing is more effective in securing rigid fixation.^14^-16)

Excessive soft tissue stripping can be avoided with retrograde intramedullary nailing, which requires no exposure of the fracture site. Accordingly, preservation of the blood circulation at the fracture site and rapid bone union can be expected. In addition, in the case of a severely comminuted fracture, reduction and rigid fixation can be achieved without additional damage to the adjacent soft tissues.17) Bezwada et al.8) compared an open reduction group with a retrograde intramedullary nailing group for the treatment of periprosthetic fractures, and obtained incomparable results in the latter group in terms of not only the operation time and hemorrhage volume but also the treatment outcomes. Although some authors point out that retrograde intramedullary nailing has limited availability due to the incompatibility of the prosthesis, sufficient space can be secured using a diamond-tip burr.18) According to some authors, rigid fixation is difficult to obtain with interlocking screws in the case of a distal fracture occurring within 20 mm of the intercondylar notch.1) However, shape memory alloy and bone cement were helpful in dealing with this problem in our study. Gliatis et al.19) reported that bone union could be obtained using retrograde intramedullary nailing in all cases of periprosthetic fractures within 3 months after the 2-year follow-up, even though revision arthroplasty using a stem was required in one case with a 35° valgus deformity. In this study, the efficacy of retrograde intramedullary nailing was demonstrated: radiographic bone union was obtained rapidly, at the 13th week on average (as late as the 15th week), and the mean range of motion at the last follow-up was 103°. Angular deformities at the fracture site were treated with a minimally invasive open reduction and cerclage fixation when a closed reduction was not successful.

This study had the following limitations: the study population was small, the study was retrospective and no proper control group was included. The pre- and postoperative functional differences could not be examined in some patients because their total knee arthroplasty had not been performed at our hospital. However, considering that the periprosthetic fracture rate was remarkably low and there have been no long-term follow-up studies on retrograde intramedullary nailing, it is believed that the current study, which included 24 to 82 months of mid-term follow-up and unprecedented postoperative functional assessment, has merit.

Retrograde intramedullary nailing for periprosthetic supracondylar fractures of the femur after total knee arthroplasty resulted in an excellent bone union rate and functional recovery. Fixation using a shape memory alloy and bone cement also appeared to be effective in promoting periprosthetic fracture healing using retrograde intramedullary nailing.

## Figures and Tables

**Table 1:**
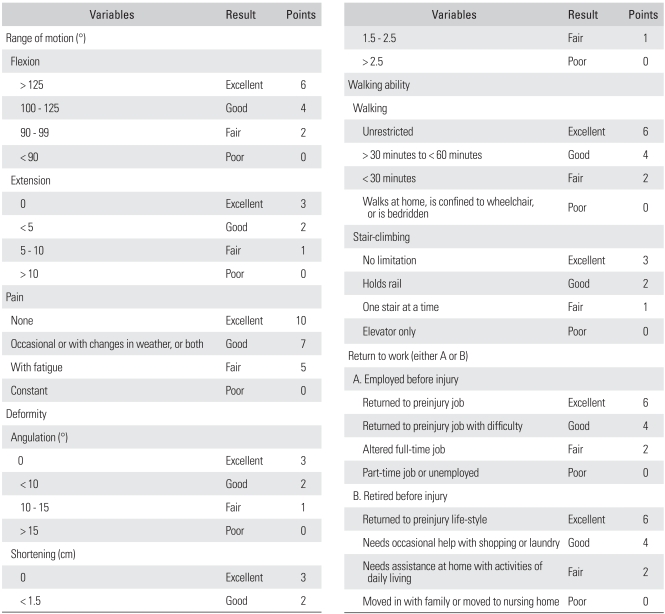
Sanders' Functional Evaluation

Excellent: 36 - 40 points, Good: 26 - 35 points, Fair: 16 - 25 points, and Poor: 0 - 15 points.

**Table 2:**
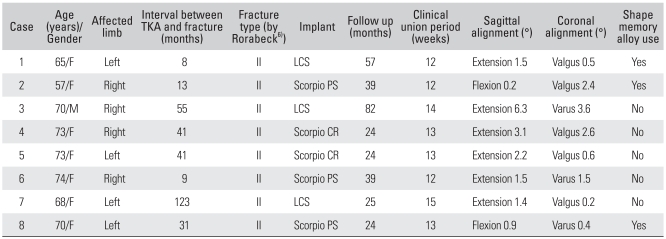
Patients' Data and Results

TKA: Total knee arthroplasty.
